# Life history traits explain the intra-seasonal abundance pattern of rare land snail species *Vertigo moulinsiana*: bridging the theory-application gap

**DOI:** 10.1038/s41598-025-10471-7

**Published:** 2025-07-09

**Authors:** Anna M. Lipińska, Adam M. Ćmiel, Dariusz Halabowski

**Affiliations:** 1 Institute of Nature Conservation, Polish Academy of Sciences, Krakow, Poland; 2 Faculty of Biology and Environmental Protection, Department of Ecology and Vertebrate Zoology, University of Lodz, Lodz, Poland

**Keywords:** Climate change impact, Conservation management, Freeze avoidance strategy, Habitat stability, Population dynamics modelling, Thermal refugia, Ecology, Conservation biology, Ecological modelling, Population dynamics, Wetlands ecology

## Abstract

*Vertigo moulinsiana*, a rare and vulnerable land snail species, faces increasing threats from climate change, particularly due to the loss of snow cover and its associated thermal buffering effects. In this study, we develop a population dynamics model to explore how life history traits, including overwintering strategies and seasonal reproduction, shape the intra-seasonal abundance patterns of *V. moulinsiana*. Using empirical data and simulated snow cover disappearance scenarios, we demonstrate the critical role of snow as an insulating layer that maintains stable subnivium (a microhabitat located at the interface between the snowpack and the ground) conditions. Without this layer, populations experience significant declines due to increased exposure to freezing temperatures and heightened mortality during snowless winters. Our findings highlight the vulnerability of *V. moulinsiana* to extreme winter conditions and emphasize the importance of integrating life history traits into ecological models. These insights provide a practical framework for conservation by identifying critical periods of vulnerability and habitat features (e.g., subnivium-like refugia) that can buffer populations against climate extremes and should be prioritized in management planning. The model is parameterized and validated using empirical data previously collected by the authors, offering a novel synthesis of life history and physiological traits in a predictive population framework.

**Supplementary Information:**

The online version contains supplementary material available at 10.1038/s41598-025-10471-7.

## Introduction

Predicting how populations and communities respond to climate change is a primary concern of global change biologists. Numerous studies have documented how climate change is already changing plant and animal species’ distribution and phenology as they attempt to adapt or track their climatic optima^1–6^. Increases in the frequency and intensity of extreme climate events (e.g. shifts in patterns of precipitation, droughts and flooding), as well as reductions in Arctic sea ice, snow cover and permafrost, are key drivers of these changes^7–9^. In particular, changes in snow cover dynamics are most pronounced in colder temperate regions, where snow-dependent species face increasing challenges due to shifting winter conditions.

Ectothermic animals are considered particularly susceptible to environmental change because their body temperatures and thus physiology vary with environmental conditions. At sub-zero temperatures, ectotherms are at risk of their body fluids freezing, and their ability to survive such conditions is referred to as cold hardiness^10^ in which supercooling (maintaining body fluids in a liquid state below their freezing point) is critical. This ability is typically assessed by measuring the supercooling point (SCP). In cool temperate and polar regions that receive substantial snowfall, winter survival for many of these organisms depends on the subnivium, a thermally stable and humid space at the snow–ground interface, which acts as a critical thermal refuge^11^. The insulating capacity of snow, resulting from its low thermal conductance, makes the subnivium essential for mitigating the freezing winter conditions. However, climate change has significantly altered the extent and duration of snow cover and frozen ground in the Northern Hemisphere^11–13^.

The duration of frozen ground without snow cover has changed most rapidly at mid-latitudes, where reductions in snow cover expose subnivium-dependent organisms to lower winter temperatures^11^. This increased exposure, coupled with more frequent freeze–thaw cycles, creates functionally colder winters for many species, altering their life history events and phenology^14^. While some species may adapt by shifting their ranges towards areas with more stable subnivium conditions^9^ others may face challenges in evolving cold tolerance due to the slow pace of such changes on a phylogenetic timescale^15^. Therefore, subnivium-dependent species are particularly vulnerable to climate-driven habitat loss and may require focused conservation efforts.

Predicting the effects of such environmental changes on subnivium-dependent species must, by necessity, rely on ecological models, as field studies documenting these impacts remain scarce, and are usually conducted in too short time periods to show the influence of climate change on the long-term population dynamics. The complexity of winter microhabitats and the challenges of studying organisms during this season have limited empirical data, leaving critical gaps in our understanding of how these species respond to rapid climatic shifts. Models provide a valuable tool to bridge this knowledge gap, offering insights into potential population dynamics and survival strategies under scenarios of reduced snow cover and increased freeze–thaw cycles. These approaches are particularly crucial for small terrestrial mollusks, where the scarcity of field data further underscores the need for predictive frameworks.

The ecological significance of Vertginid gastropods lies in their role as indicators of habitat quality, especially in the context of conservation and biodiversity assessment^16–19^. Nevertheless, their diversity has declined at a significant rate over the last decade (e.g^20^). Despite numerous studies on the species’ life history traits^21^ a comprehensive synthesis linking these traits to population dynamics under changing environmental conditions is still lacking, and as a result, their conservation poses unresolved challenges. In fact, only a few studies have provided empirical data on population dynamics or demography of these species^21–26^ despite the fact that basic methodological standards for life history trait research on *Vertigo* spp. were proposed decades ago by Pokryszko^20,27^ and Myzyk^21,26^. This gap in knowledge hampers our ability to assess population viability, understand long-term trends, and anticipate responses to environmental pressures such as habitat alteration or climate change.

In this study, we propose a simple population dynamics model for *Vertigo moulinsiana*, a rare and protected species in Europe. The model is based on key life history traits^21,28^ including age at maturation, seasonality and frequency of reproduction, stage-specific survival, and overwintering as adults. While the overall life-cycle structure reflects general patterns observed in molluscan demography, most parameter values were drawn from empirical data collected by Myzyk^21,28^. The division into three adult age classes reflects differences in reproductive output and survival between first-time and older reproducers. By integrating these traits, the model explores how population size and structure change over time in response to climate-related factors, such as the disappearance of snow cover.

Population dynamics models allow for the exploration of changes in population size and structure over time by integrating key demographic processes such as birth, death, immigration, and emigration^29^. By simulating multiple ecological parameters, they provide a powerful framework for examining age-structured responses to environmental change under a range of climate scenarios^30^.

This study addresses whether a simple process-based population model, built on basic life history parameters, can be used to accurately reconstruct the intra-seasonal abundance dynamics of the rare and threatened land snail *Vertigo moulinsiana* under conditions of limited detectability. The model integrates traits such as seasonal reproduction, overwintering strategy, and age-specific mortality, and is validated against field observations. We further examine whether this model can be extended to simulate population responses under alternative climatic scenarios (e.g. snowless winters), providing a tool to anticipate short-term climate-related risks and inform conservation planning for species with narrow ecological niches and complex life cycles. The model is based entirely on empirical datasets previously collected by the authors, including demographic parameters, density estimates, and cold tolerance thresholds. While some components of these datasets have been presented in earlier studies addressing different questions, this is the first time they have been integrated into a population dynamics modelling framework for this species.

## Materials and methods

### Study species

*Vertigo moulinsiana* is a minute land snail (with a shell 2.7 mm high and 1.6 mm wide) that has been recognised as vulnerable throughout Europe^31^ and is listed in Appendix II of the Habitats Directive. The snail inhabits open wetlands in lowland areas, characterized by high water and soil calcium levels^25^ and is sparsely distributed in Central Europe.

The main factor responsible for the occurrence of this species is a level of water that fluctuates around ground level^17,24,32,33^. *V. moulinsiana* snails live over a vertical range and can be found high up on vegetation at certain times of the year^34^. With the onset of winter, *V. moulinsiana* overwinters on sedge tussocks^27,28,32^. Adults of *V. moulinsiana* usually overwinter on plants, whereas young snails, more fragile to desiccation, do so in the litter^35,36^. The overwintering of *V. moulinsiana* has already been discussed in several studies^28,35–38^ and it is known that winter survival in *V. moulinsiana* is relatively high - between 60 and 73% - and is not dependent on the habitat type^28^.

During the winter, the snails occur in thermally buffered microhabitats beneath a canopy of dry vegetation and snow^39^. It is very likely that *V. moulinsiana* employs a freeze avoidance strategy and that the formation of ice in their tissues is lethal to *V. moulinsiana* snails^39^. The mean supercooling point (SCP; the temperature at which ice crystals begin to form in an individual’s body fluids) was found to be at -9.9 °C in winter, with a wide range between the lowest and the highest measurement (-6.3 to -15 °C in winter). Mean SCP did not differ significantly between young and adult snails^39^.

The general species description is given in^27^ with more detailed demographic and reproductive data in^21^,. *V moulinsiana* is hermaphrodite, mostly self-fertilising^40^. A typical population consists of 3 overlapping generations due to a mean life span of individuals equal to 15 months, but most individuals live for 10–15 months^32^. The mortality of adult individuals between consecutive months ranges between 10 to 15%^21^. Each individual lays a mean of 19 eggs during the season between May and August, and the laid eggs hatch from June to September^21^. The mean time of reaching maturity for young individuals is 99 days from hatching^21^. Most of the young individuals reach maturity in the following season, while 10–15% reach maturity in the season of hatching, usually when the breeding period is finished^21^. Only juvenile and adult individuals overwinter; egg overwintering has not been observed^28^.

The very characteristic trait of this species is the large between- and within-seasonal fluctuations of the population abundance^23,32^for which mechanism has not been explained to date.

### Population dynamic model - description

**Fig. 1:**
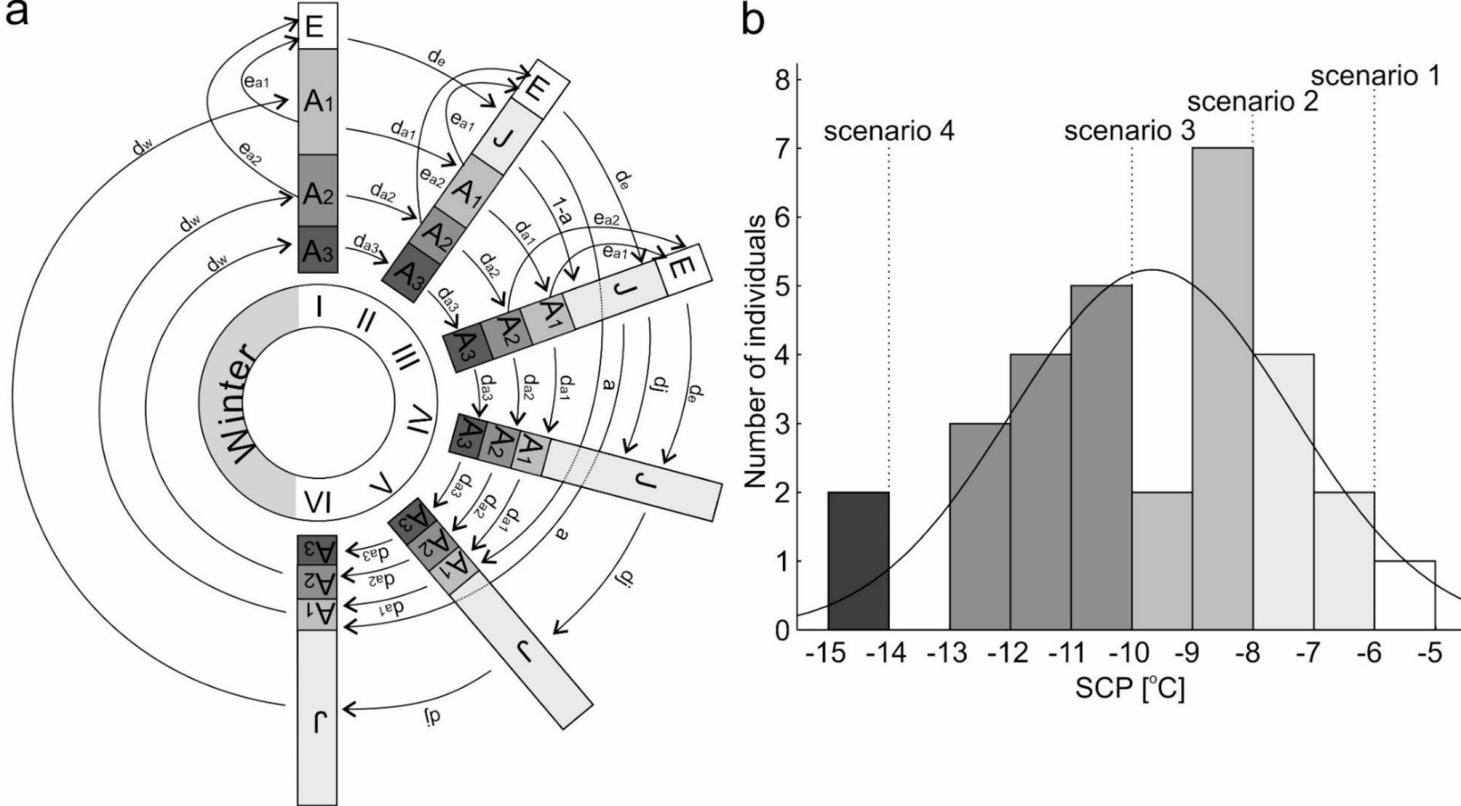
(**a** ) conceptual diagram of the model. E – eggs, J – juveniles, A_1_ – adult individuals at age 1, A_2_ – adult individuals at age 2, A_3_ – adult individuals at age 3, *d*_*w*_ – winter survival rate, *d*_*a1*_ – survival rate of adults at age 1, *d*_*a2*_ – survival rate of adults at age 2, *d*_*a3*_ – survival rate of adults at age 3, *d*_*e*_ – eggs hatching rate, *e*_*1*_ – number of eggs laid by adults at age 1, *e*_*2*_ – number of eggs laid by adults at age 2, *e*_*3*_ – number of eggs laid by adults at age 3, *d*_*j*_ *–* survival rate of juveniles, *a* – recruitment rate of juveniles. (**b**) histogram of supercooling point (SCP) for *Vertigo moulinsiana*. Dashed lines indicate SCP thresholds corresponding to four modelled climate scenarios, illustrating potential impacts of cold stress on population survival (data from^35^).

The constructed deterministic, discrete time model simulates age structured *V. moulinsiana* population with five different life stages: eggs, juveniles (newly hatched immature individuals) and adult individuals in one of three possible age classes: 1 (newly matured individuals before their first overwintering period, 2 (mature individuals after one overwintering period) and 3 (mature individuals after two overwintering periods). Each modelled year (hereinafter referred to as the season) consist of six time steps (months) corresponding to the activity period of *V. moulinsiana* (from May to October). At each time step, different survival rates describe mortality processes for the cohorts, while the reproduction modelled population is described by number of eggs laid through sexual reproduction by adult individuals and eggs hatching rate. Mortality during the overwintering period (from December to May) is described by the winter survival rate. The conceptual diagram of the model was presented at Fig. 1a.

Let us denote the number of individuals in month i of season j by *N(i*,* j)*. Let *N*_*1*_*(i*,* j)* be the number of adult individuals at age 1, *N*_*2*_*(i*,* j)* be the number of adult individuals at age 2, *N*_*3*_*(i*,* j)* be the number of adult individuals at age 3. Let *E*_*1*_*(i*,* j)* be the number of eggs laid by adult individuals at age 1, *E*_*2*_*(i*,* j)* be the number of eggs laid by adult individuals at age 2. Let us denote the ratio of hatching eggs by *d*_*e*_. The number of juvenile individuals in month *i* of season *j* is denoted by *J(i*,* j)*. Let us denote the survival between successive months for individuals at a given age by *d*_*a1*_, *d*_*a2*_, *d*_*a3*_ and the survival of juveniles by *d*_*j*_, and the winter survival of all individuals (both juveniles and adults) by *d*_*w*_.

The number of eggs laid in a given month *i* of season *j* by adult individuals at age 1 and 2 is given by:

1 $$\:E\left(i,j\right)={N}_{1}\left(i,j\right){e}_{1}\left(i\right)+{N}_{2}\left(i,j\right){e}_{2}\left(i\right)$$

where *e*_*1*_*(i)* is the number of eggs laid by individuals at age 1 and *e*_*2*_*(i)* is the number of eggs laid by individuals at age 2 in a given month *i.*

The number of juvenile individuals hatched in a given month *i* from the eggs laid in previous month (*i*-1) is given by:

2 $$\:J\left(i,j\right)=\left\{\begin{array}{c}0\:\:\:for\:\:\:i=1\\\:E\left(i-1,j\right){d}_{e}\:\:\:for\:\:\:i=2\\\:J\left(i-1,\:j\right){d}_{j}-\left(1-a\right)J\left(i-3,j\right){d}_{j}+E\left(i-1,j\right){d}_{e}\:\:for\:\:\:i=5\\\:J\left(i-1,\:j\right){d}_{j}-\left(1-a\right)J\left(i-3,j\right){d}_{j}+E\left(i-1,j\right){d}_{e}\:\:for\:\:\:i=6\\\:J\left(i-1,\:j\right){d}_{j}+E\left(i-1,j\right){d}_{e},\:\:\:\:otherwise\end{array}\right.$$

The length of the juveniles’ period of growth depends on the month of hatching. The ratio of recruited juveniles is given by *a*.

The number of individuals in a given age class in a given month *i* given by:

3 $$\:{N}_{1}\left(i,j\right)=\left\{\begin{array}{c}J\left(i+5,j-1\right){d}_{w}\:\:\:\:for\:\:\:i=1\\\:{N}_{1}\left(i-1,\:j\right){d}_{a1}+aJ\left(i-3,j\right){d}_{j}\:\:\:for\:\:\:i=5\\\:{N}_{1}\left(i-1,\:j\right){d}_{a1}+aJ\left(i-3,j\right){d}_{j}\:\:\:for\:\:\:i=6\\\:{N}_{1}\left(i-1,\:j\right){d}_{a1}\:\:\:\:\:otherwise\end{array}\right.$$

4 $$\:{N}_{2}\left(i,\:j\right)=\left\{\begin{array}{c}{N}_{1}\left(i+5,j-1\right){d}_{w}\:\:\:for\:\:i=1\\\:{N}_{2}\left(i-1,j\right){d}_{a2}\:\:\:\:\:otherwise\end{array}\right.$$

5 $$\:{N}_{3}\left(i,\:j\right)=\left\{\begin{array}{c}{N}_{2}\left(i+5,j-1\right){d}_{w}\:\:\:\:for\:\:\:i=1\\\:{N}_{3}\left(i-1,j\right){d}_{a3}\:\:\:\:\:otherwise\end{array}\right.$$

The total number of individuals in a given month *i* of a given season *j* is a sum of juveniles and adults at each age class and is given by:

6 $$\:N\left(i,j\right)={N}_{1}\left(i,j\right)+{N}_{2}\left(i,j\right)+{N}_{3}\left(i,j\right)+J(i,j)$$

The model was programmed in the SciLab 6.0 numerical computational package. Data used for the model testing were collected in the field, from the study area in the Inland Delta of the Nida River (50°34’30”N, 20°31’27”E; details in^25^), between May and October 2008–2010. The model parameters were set at the values recorded both in laboratory^21^ and field studies on *Vertigo moulinsiana*^28^ which were presented in the Supplementary Materials (Table S1). Model verification, testing and basic sensitivity analysis were described and presented in the Supplementary Materials (model verification and testing: Table S2, Fig. S1-S3; sensitivity analysis: Table S3-S5).

### Snow cover disappearance scenarios

Based on earlier study^39^ where the SCP of snail body fluids was determined, we modelled four scenarios of snow cover disappearance, each differing in the minimum air temperature (t_min_) occurring during the winter: (1) t_min_ = -5.5^o^C, corresponding to the maximum supercooling point measured for all individuals and to the minimum air temperature measured in the field in November; (2) t_min_ = -8^o^C, corresponding to the median supercooling point measured for juvenile individuals and to the minimum air temperature measured in the field in December; (3) t_min_ = -10^o^C, corresponding to the median supercooling point measured for all individuals and (4) t_min_ = -14^o^C, corresponding to the minimum supercooling point measured for all individuals and to the minimum air temperature measured in January. The occurrence of given minimum air temperature, without buffering snow cover layer, results in increased winter mortality of individuals, due to lethal ice crystallisation in their tissues (3% mortality at -6^o^C, 23% mortality at -8^o^C, 50% mortality at -10^o^C and 95% mortality at -14^o^C; Fig. 1b). Thus the value of the winter survival parameter (*d*_*w*_) was decreased from 0.7 to 0.68 in scenario 1, 0.54 in scenario 2, 0.35 in scenario 3 and 0.04 in scenario 4. Also, one additional simulation (Scenario “0”), using unchanged initial values of model parameters, was performed.

To show the differences between scenarios, the mean final population size, minimum population size and maximum population size during the last modelled season (*j* = 20) was calculated. Also, for each scenario, time to extinction of population was determined, and mean seasonal population growth rate (λ) was calculated using the formula:

7 $$\:\lambda\:=\frac{{\stackrel{-}{N}}_{20}-{\stackrel{-}{N}}_{1}}{{\stackrel{-}{N}}_{1}}\cdot\:100\%$$

where $\:{\stackrel{-}{N}}_{1}$is a mean population size during the first modelled season (*j* = 1) and $\:{\stackrel{-}{N}}_{20}$ is a mean population size during the last modelled season (*j* = 20).

## Results

### Snow cover disappearance scenarios

**Fig. 2:**
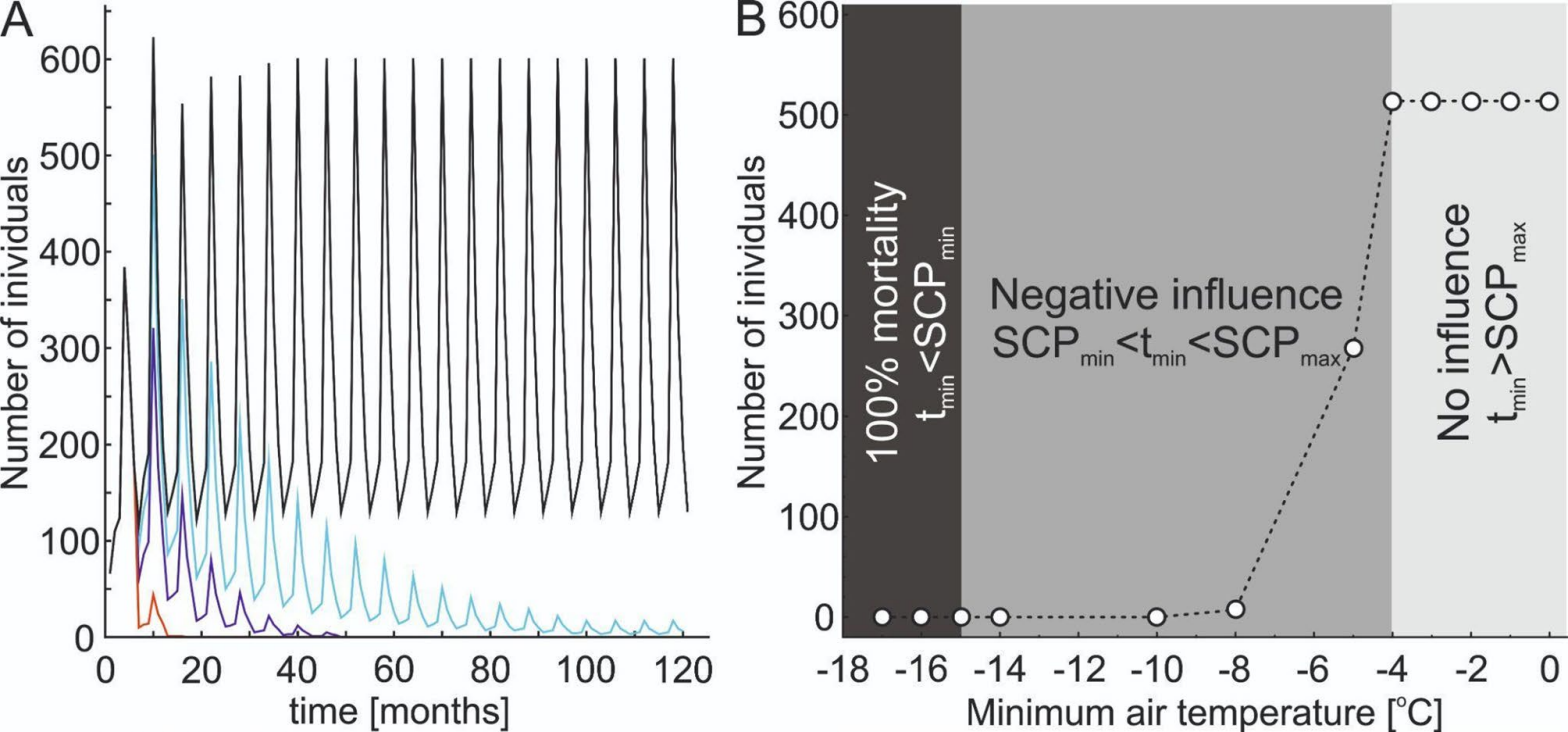
(**a**) population size during simulated 20 consecutive seasons (120 months in total) in four adopted scenarios of snowless winters: (1) with minimum air temperature a -5.5^o^C (black line), (2) with minimum air temperature at -8^o^C (green line), (3) with minimum air temperature at -10^o^C (dark blue line) and (4) with minimum air temperature at -15^o^C (red line). (**b**) the influence of minimum air temperature during snowless winters on final population size after 20 seasons (12 months). t_min_ – minimum air temperature occurring during the winter, SCP_min_ – minimum Supercooling temperature value obtained for *Vertigo moulinsiana*, SCP_max_ – maximum Supercooling temperature value obtained for *Vertigo moulinsiana*.

The results of simulated scenarios showed that a snowless winter may have a very negative influence on the snail’s population size, depending on the minimum air temperature occurring during the winter (Fig, 2 a, b). In scenario 1, which assumed the minimum air temperature at -5.5^o^C, which is only slightly lower than maximum SCP, over four times decrease in mean annual population growth rate, and almost two times lower final population size were observed, compared to the “0” scenario (stagnating snow cover; Table 1). Nevertheless, in this scenario, population size was stable, showing regular, within-seasonal fluctuations (Fig. 2a).

**Table 1:** Basic statistics of simulated scenarios (1–4) and scenario “0” (simulation using unchanged initial values of model parameters) of *V. moulinsiana* winter survival.

Scenario	Mean final population size (t = 120)	SD	Minimum population size	Maximum population size	Time to extinction [months]	Mean annual population growth rate (λ)
“0”	514	343.7	249	1156	-	1.4%
1	267.5	178.6	130	601	-	0.33%
2	7.3	5.2	3	17	-	-0.8%
3	0	-	0	0	49	-2.1%
4	0	-	0	0	17	-5.5%

In scenario 2, which assumed the minimum air temperature at -8^o^C, mean annual population growth rate was negative, whereas the mean final population size was very low and ca. 70 times lower, compared to the “0” scenario (Table 1). Moreover, in this scenario, population size was decreasing for ca. 100 months, but after that time, it stabilized at low level (Fig. 2a).

In scenarios 3 and 4, which assumed the minimum air temperature at -10^o^C and -14^o^C accordingly, mean annual population growth rates were negative (Table 1; Fig. 2) and as a result, the population size rapidly decreased, and, in both scenarios, populations became extinct after 49 months (during 8th season; scenario 3), and after 17 months (during 2nd season; scenario 4).

## Discussion

The simplicity and effectiveness of mathematical models make them valuable tools for understanding the population dynamics of species like *V. moulinsiana*. These models, based on straightforward life history data, align well with field observations and provide a framework for predicting future population trends. The model developed in this study not only formalizes life history traits but also offers valuable insights into the population dynamics of gastropods, a group for which field studies are often logistically challenging. In contrast, basic life history traits can be more easily quantified under controlled conditions^21,27^ making modelling an essential tool for both research and conservation planning.

Although mathematical modelling has been widely used in population ecology, relatively few population models have been developed for gastropods. The majority of existing models focus on slugs, primarily because of their status as agricultural pests and the associated need to control their populations (e.g.:^41–43^). Similarly, modelling efforts have often targeted non-native or invasive gastropod species (e.g.:^44,45^), driven by concerns about their ecological impacts and the necessity of population suppression. As a result, native and non-pest gastropods, particularly those of conservation concern, remain largely underrepresented in demographic modelling studies. This highlights a critical gap in the literature and underscores the value of developing models for such species, not for the purpose of control, but to support long-term conservation and management planning.

Our model highlights the importance of specific life history traits in maintaining population stability. Given the scarcity of demographic data for most threatened species, particularly gastropods^46^ categorizing species based on their life history traits offers a practical and effective framework. This approach assumes that demographic patterns largely conform to general trends, offering a useful predictive tool for conservationists. In addition, our model also reveals an intriguing aspect of *V. moulinsiana* populations, namely a sharp population decline during autumn. While the species demonstrates mechanisms promoting resilience during spring and summer, such as a large influx of juveniles, it lacks compensatory strategies in the later part of the season. The absence of new egg clutches or juveniles following the peak leads to a marked decline, as observed by Myzyk^21^. This seasonal “die-off” is likely a direct consequence of life history traits. Our study emphasizes that limited-dispersal species like *V. moulinsiana* are particularly sensitive to environmental changes, which can lead to fluctuations in their intra-seasonal abundance.

According to current climate change projections^11,47^ mid-latitude regions are likely to experience a phase where winters become increasingly snowless, while sub-zero air and soil temperatures persist. For subnivium-dependent species such as *V. moulinsiana*, this sequence of environmental change may be particularly critical, as the loss of the insulating snow layer exposes individuals to lethal freeze–thaw cycles. This period of decoupling between snow cover and frost conditions may result in range contractions or even local extinctions, especially in areas where alternative thermal refuges are unavailable. Our modelling scenarios are therefore relevant for anticipating short- to mid-term responses of cold-sensitive species during this vulnerable transition phase. Importantly, our scenarios do not imply a linear relationship between climate warming and declining frost occurrence. Rather, they are designed to capture a critical transitional window — before extreme cold events become infrequent — during which the loss of snow cover may paradoxically increase cold exposure. This phase may vary in duration and intensity depending on local climate trajectories, and while it may not represent long-term future conditions, it likely reflects an ecologically significant near-future challenge for subnivium-dependent species. a microhabitat located at the interface between the snowpack and the ground.

While direct real-world analogues of completely snow-free but frosty winters are currently limited, occasional winters in lowland regions of Central and Eastern Europe already exhibit characteristics resembling our modelled scenarios. Such conditions, although infrequent, may become increasingly common and offer a glimpse into likely near-future winter environments for subnivium-dependent species. Simulated scenarios of snowless winters further emphasize the critical role of snow cover in the survival and long-term stability of *V. moulinsiana* populations. Even a slight reduction in minimum winter temperatures (Scenario 1) caused a fourfold decrease in mean annual population growth rates and halved the final population size compared to the baseline scenario with stable snow cover. Nonetheless, the population remained stable, with regular seasonal fluctuations, indicating some resilience to moderately suboptimal conditions. In Scenario 2 (-8 °C minimum), the mean annual population growth rate turned negative, and the final population size dropped to 70 times lower than the baseline scenario. Although the population stabilized at a low level after 100 months, such a decline signals vulnerability to sustained stress. Scenarios 3 and 4 (-10 °C and -14 °C minimums, respectively) showed rapid population declines and eventual extinction. Populations disappeared within 49 months (Scenario 3) and just 17 months (Scenario 4). These findings highlight the inability of *V. moulinsiana* to endure severe frost conditions without the protective buffer of snow cover.

Snow cover provides a critical insulating layer during winter, composed of decomposing plant material, mulch, and snow, maintaining stable subnivium conditions critical for *V. moulinsiana* survival^39^. This stable microclimate protects snails from extreme temperature fluctuations and has been widely documented as essential for other organisms^48–51^. In addition, dense litter and vegetation cover significantly buffer ground-level temperatures, often reducing temperature minima by several degrees compared to exposed soil (e.g^52,53^). These structures also provide protection from predators and maintain humidity levels, which are critical for the activity and reproduction of land snails^33,50,53^. This buffering effect may reduce the need for costly physiological adjustments, such as lowering the SCP (supercooling point) to extreme levels^54^. However, individuals with SCPs as low as -15 °C may provide a safeguard for population persistence in the absence of adequate shelters. While this variation in SCP^39^ suggests potential differences in cold tolerance among individuals, the underlying mechanisms remain unclear and may result from genetic differences, environmental influences, or a combination of both. Further research is needed to determine whether this variability reflects phenotypic plasticity or other adaptive processes. Regardless of its origin, such variability is unlikely to fully mitigate the negative effects of sustained environmental stress, particularly under scenarios with prolonged or extreme snow cover loss. Especially because beneath the snow, temperatures can range from 0 °C to 2 °C, even when air temperatures above the snow are 4 °C to 22 °C lower^55^. Given that vegetation structure contributes significantly to litter formation and snow retention, habitat degradation through vegetation loss could indirectly affect the availability and quality of subnivium refuges, further increasing the vulnerability of *V. moulinsiana* to climate extremes.

In this study we present a general mathematical framework of the consequences of snow cover disappearance. However, one should be aware that the model was parameterized, calibrated and tested using data obtained from the certain *V. moulinsiana* population, whereas life history traits in Gastropods may vary within and among species, as well as between populations of the same species. The constructed deterministic model is very general, e.g. mortality in each life stage is described by one parameter, without identifying the specific causes of mortality (e.g. predation, food availability). Also, all parameters are assumed to be constant throughout the whole modelling exercise, which of course should be considered as unrealistic, but simultaneously enables to show the influence of a change in one given parameter (winter survival rate) in a very simple way. Moreover, very little or nothing is known about energetic costs of lowering the freezing temperature of body fluids and related trade-offs, which also forces the model to be simple. Even so, the results obtained with the model, which formulates the problem explicitly, identifies some knowledge gaps and addresses some hitherto unidentified questions.

Overall, our results highlight the ecological importance of snow cover as a thermal buffer, maintaining stable subnivium conditions critical for *V. moulinsiana* survival. Without this insulating layer, snails are exposed to lethal freezing temperatures, resulting in significant mortality. The variation in SCP observed among individuals indicates differences in cold tolerance, though the underlying mechanisms remain unclear. Conservation efforts should focus on preserving microhabitats that buffer against extreme conditions and mitigating the impacts of snow loss to safeguard the long-term survival of this vulnerable species. In particular, identifying and protecting climatic refugia—such as shaded depressions, peatlands with thick litter layers, or densely vegetated microsites—may help maintain subnivium-like conditions in the absence of snow cover. These natural shelters can offer localized thermal and humidity stability, acting as functional analogues of the snow layer and providing critical overwintering habitat. Prioritizing such areas in conservation planning offers a realistic, habitat-based approach to mitigating climate-driven risks.

Additionally, conservation efforts could include active habitat management, such as placing layers of cut vegetation or hay in *V. moulinsiana* habitats during late autumn to artificially enhance insulation and reduce exposure to lethal winter temperatures. Our model results suggest that such simple, low-cost interventions can mitigate the negative effects of snow cover loss and may significantly improve winter survival. These measures are particularly important for protecting juvenile individuals, which are more vulnerable to freezing due to their thin, incompletely developed shells and higher mortality rates compared to adults. Since most adults die after one year, it is primarily the juvenile cohort that overwinters and thus plays a critical role in population persistence. Although not yet tested in this species, such artificial shelters may functionally replace the thermal buffering provided by snow and contribute to overwinter survival. Translocation to areas with more stable subnivium conditions may also be considered in extreme cases, provided that habitat suitability and genetic compatibility are carefully evaluated. These alternative interventions, in combination with habitat protection, offer a broader toolbox for conserving cold-sensitive species under climate change.

Looking ahead, adapting this modelling framework to a broader range of gastropods, including species with differing ecological traits and geographical distributions, would enhance its utility. Future work could also integrate genetic or physiological data to capture intraspecific variation in responses to environmental change, thereby increasing the model’s relevance for real-world conservation scenarios.

## Electronic supplementary material

Below is the link to the electronic supplementary material.

Supplementary Material 1

## Data Availability

The datasets generated and analysed during the current study are available in the GitHub repository, https://github.com/CmielAM/Vertigo-Life-history-traits/tree/main.
