# Supplementary material for: Life history traits explain the intra-seasonal abundance pattern of rare land snail species Vertigo moulinsiana: bridging the theory-application gap
Source: Sci Rep. 2025 Jul 9;15:24737. doi: 10.1038/s41598-025-10471-7 (PMC12241451; doi:10.1038/s41598-025-10471-7)
Supplement: Supplementary file 1 — Supplementary Material 1 [file 41598_2025_10471_MOESM1_ESM.docx]

**Supplementary Materials for**

**Life history traits explain the intra-seasonal abundance pattern of rare land snail species *Vertigo moulinsiana*: bridging the theory-application gap**

Anna M. Lipińska^1,*^, Adam M. Ćmiel^1^, Dariusz Halabowski^2^

^1^ Institute of Nature Conservation, Polish Academy of Sciences, Krakow, Poland;

^2^ University of Lodz, Faculty of Biology and Environmental Protection, Department of Ecology and Vertebrate Zoology, Lodz, Poland;

* Correspondence: lipinska@iop.krakow.pl

The conceptual diagram of the model was presented at Fig. 1a. in the main text. The model was programmed in the SciLab 6.0 numerical computational package. Data used for the model testing were collected in the field, from the study area in the Inland Delta of the Nida River (50°34’30”N, 20°31’27”E; details in^25^), between May and October 2008-2010. The model parameters were set at the values recorded both in laboratory^21^ and field studies on *Vertigo moulinsiana*^28^ (Table S1).

**Table S1**. Parameter values for *Vertigo moulinsiana* model based on the laboratory and field study.

| Parameters | Symbol | Value | Source |
| --- | --- | --- | --- |
| Winter survival rate | *d_w_* | 0.7 | ^28^ |
| Survival rate of adults at age 1 | *d_a1_* | 0.92 | ^21^ |
| Survival rate of adults at age 2 | *d_a2_* | 0.82 | ^21^ |
| Survival rate of adults at age 3 | *d_a3_* | 0.7 | ^21^ |
| Eggs hatching rate | *d_e_* | 0.4 | ^21^ |
| Number of eggs laid by adults at age 1 during 1^st^ month of the breeding season | *e_1_(1)* | 1 | ^21^ |
| Number of eggs laid by adults at age 1 during 2^nd^ month of the breeding season | *e_1_(2)* | 1 | ^21^ |
| Number of eggs laid by adults at age 1 during 3^rd^ month of the breeding season | *e_1_(3)* | 10 | ^21^ |
| Number of eggs laid by adults at age 2 during 1^st^ month of the breeding season | *e_2_(1)* | 2 | ^21^ |
| Number of eggs laid by adults at age 2 during 2^nd^ month of the breeding season | *e_2_(2)* | 2 | ^21^ |
| Number of eggs laid by adults at age 2 during 3^rd^ month of the breeding season | *e_2_(3)* | 15 | ^21^ |
| Survival rate of juveniles | *d_j_* | 0.5 | ^21^ |
| Recruitment rate of juveniles | *a* | 0.15 | ^21^ |

***Population dynamic within seasonal pattern***

Within each of the seasons the population dynamics show a repeatable pattern (Fig. S1): the sum of individuals is the lowest in the first month on the season (May), then, it increases due to hatching of the young snails from small spring clutches. After that, the number of individuals is more stable (until third month; July), but is still growing due to the hatching of juveniles from the eggs laid by a small number of adults from the onset of the season. The population number increases rapidly in the fourth month of the season (August; ca. 4-fold increase in number) which is determined by the large number of juveniles hatched from large summer clutches. Later, during fifth and sixth month of the season (September and October), the population number decreases rapidly because in late summer the summer juveniles have very large, although stable, mortality (50% from month to month). Moreover, adult individuals suffer increased mortality from 8% (adult individuals at age 1) to 30% (adult individuals at age 3).

**

**

**Fig. S1.** Within-seasonal pattern of modelled *Vertigo moulinsiana* population with the age structure of the population.

**Model verification and testing**

The initial number of individuals was set to 166 (100 individuals at age 1, 63 individuals at age 2 and 3 individuals at age 3). The model was run for 3 consecutive seasons (18 months in total) and its results were compared to the observed values of *Vertigo moulinsiana* population density (^28^,Table S2, Fig. S2).

Levene’s test showed that the homogeneity of variance assumption was fulfilled (Levene’s test; F=0.21, p=0.6462), and further statistical tests showed that observed and predicted population densities are highly correlated (Pearson correlation; r=0.73, p=0.0008, Fig. S3), and that the differences between observed and predicted population density (Table S2) are not significant (t test for dependent samples; df=16, t=-0.95, p=0.3580). Coefficient of determination (R^2^), calculated using the linear regression showed that that R^2^=0.54.





**Fig. S2.** Testing of *Vertigo moulinsiana* model – population density predicted by the model vs population density observed in the field during study between 2008 and 2010.

**Table S2.** Density of *Vertigo moulinsiana* snails observed in the field during 2008-2010 period and density predicted by the tested model. Data on population density in August 2010 were not collected due to a catastrophic flood, which occurred on the study site.

| Year | Month | Observed population  density [ind./m^2^]  (surce: ^28^) | Predicted population  density [ind./m^2^] |
| --- | --- | --- | --- |
| 2008 | May | 0.87 | 0.87 |
|  | June | 1.22 | 1.24 |
|  | July | 1.21 | 1.35 |
|  | August | 2.1 | 3.48 |
|  | September | 0.35 | 2.34 |
|  | October | 0.33 | 1.47 |
| 2009 | May | 1.24 | 1.25 |
|  | June | 2.82 | 1.92 |
|  | July | 2.84 | 1.95 |
|  | August | 4.38 | 6.45 |
|  | September | 2.34 | 3.63 |
|  | October | 1.32 | 2.13 |
| 2010 | May | 1.17 | 1.65 |
|  | June | 1.56 | 2.7 |
|  | July | 6 | 3 |
|  | August | - | 10.2 |
|  | September | 6.21 | 5.64 |
|  | October | 3.72 | 3.48 |





**Fig. S3.** Scatterplot of population density of *Vertigo moulinsiana* snails observed in the field during 2008-2010 period and population density predicted by the tested model.

**Sensitivity analysis**

Sensitivity analysis were performed using the one-factor-at-a-time (OAT) method, by moving one input variable, while keeping others at their nominal values, then, returning the examined variable to its nominal value, and repeating the procedure for each of the other input variables in the same way.

To assess the influence of the survival parameters of adults at each age class (*d_a1_*, *d_a2_*, *d_a3_*), the survival rate of juveniles (*d_j_*), egg hatching rate (*d_e_*) and winter survival rate (*d_w_*) on: 1) the mean monthly population growth rate, 2) on the number of individuals in the population peak (in fourth month) of the season and 3) on the mean final population size after 20 modelled seasons (corresponding to the 20 seasons long simulations presented in the main text), model runs were carried out for each parameter, changing from 0 to 1 with a step of 0.1, while the other parameters were fixed at their default (starting) values (listed in table S1). The results were presented in Tables S3-S5.

The results showed, that the survival rate of juveniles (*dj*) has the highest influence on the population growth rate (Table S3). The second most important parameter influencing population growth rate in the following season was the eggs hatching rate (*de*), while the other modelled parameters were less important (Table S3).

The eggs hatching rate (*de*) is the most important factor influencing the number of individuals in the population during population peak, the survival rate of juveniles (*dj*) was the second most important factor, whereas the influence of other factors is similar to each other and ca. more than 2 times lower than the influence of the survival rate of juveniles (Table S4).

The survival rate of juveniles (*dj*) has the highest influence on the final population size after 20 seasons (Table S5), while the eggs hatching rate (*de*) was the second most important parameter influencing the final population size after 20 seasons. The other modelled parameters were far less important and compared to the influence of the survival rate of juveniles eggs hatching rate , their influence is almost negligible (Table S5).

**Table S3.** Values of mean monthly population growth rate (λ) depending on the values of model parameters obtained during sensitivity analysis using the one-factor-at-a-time method. *d_j_ –* survival rate of juveniles, *d_e_* – eggs hatching rate, *d_w_* – winter survival rate, *d_a1_* – survival rate of adults at age 1, *d_a2_* – survival rate of adults at age 2, *d_a3_* – survival rate of adults at age 3.

| Parameter value | Parameter | | | | | |
| --- | --- | --- | --- | --- | --- | --- |
|  | *d_j_* | *d_e_* | *d_w_* | *d_a1_* | *d_a2_* | *d_a3_* |
| 0 | -0.08 | -0.08 | -0.14 | -0.05 | -0.03 | 0.18 |
| 0.1 | -0.04 | -0.03 | -0.05 | -0.05 | -0.03 | 0.18 |
| 0.2 | -0.03 | -0.03 | -0.03 | -0.04 | -0.02 | 0.18 |
| 0.3 | -0.03 | -0.02 | -0.02 | -0.04 | -0.01 | 0.18 |
| 0.4 | 0.00 | 0.01 | -0.01 | -0.03 | 0.01 | 0.18 |
| 0.5 | 0.18 | 0.07 | 0.02 | -0.03 | 0.04 | 0.18 |
| 0.6 | 0.98 | 0.18 | 0.08 | -0.03 | 0.07 | 0.18 |
| 0.7 | 4.03 | 0.36 | 0.18 | -0.02 | 0.11 | 0.18 |
| 0.8 | 14.39 | 0.64 | 0.33 | 0.02 | 0.17 | 0.18 |
| 0.9 | 46.05 | 1.05 | 0.55 | 0.14 | 0.23 | 0.18 |
| 1 | 135.28 | 1.66 | 0.85 | 0.47 | 0.31 | 0.18 |

**Table S4.** Number of individuals during the population peak (in fourth month of the season) depending on the values of model parameters obtained during sensitivity analysis using the one-factor-at-a-time method. *d_j_ –* survival rate of juveniles, *d_e_* – eggs hatching rate, *d_w_* – winter survival rate, *d_a1_* – survival rate of adults at age 1, *d_a2_* – survival rate of adults at age 2, *d_a3_* – survival rate of adults at age 3.

| Parameter value | Parameter | | | | | |
| --- | --- | --- | --- | --- | --- | --- |
|  | *d_j_* | *d_e_* | *d_w_* | *d_a1_* | *d_a2_* | *d_a3_* |
| 0 | 290 | 55 | 0 | 0 | 24 | 548 |
| 0.1 | 334 | 159 | 79 | 54 | 43 | 548 |
| 0.2 | 362 | 274 | 157 | 90 | 69 | 548 |
| 0.3 | 398 | 394 | 243 | 143 | 95 | 549 |
| 0.4 | 464 | 576 | 346 | 195 | 152 | 549 |
| 0.5 | 554 | 743 | 398 | 258 | 221 | 551 |
| 0.6 | 680 | 954 | 481 | 313 | 313 | 552 |
| 0.7 | 845 | 1190 | 554 | 387 | 406 | 554 |
| 0.8 | 1038 | 1449 | 644 | 462 | 529 | 557 |
| 0.9 | 1249 | 1722 | 743 | 548 | 667 | 561 |
| 1 | 1575 | 2028 | 793 | 629 | 843 | 565 |

**Table S5.** Mean final population size after 20 modelled seasons depending on the values of model parameters obtained during sensitivity analysis using the one-factor-at-a-time method. *d_j_ –* survival rate of juveniles, *d_e_* – eggs hatching rate, *d_w_* – winter survival rate, *d_a1_* – survival rate of adults at age 1, *d_a2_* – survival rate of adults at age 2, *d_a3_* – survival rate of adults at age 3.

| Parameter value | Parameter | | | | | |
| --- | --- | --- | --- | --- | --- | --- |
|  | *d_j_* | *d_e_* | *d_w_* | *d_a1_* | *d_a2_* | *d_a3_* |
| 0 | 0 | 0 | 0 | 0 | 490 | 498 |
| 0.1 | 0 | 0 | 0 | 0 | 494 | 499 |
| 0.2 | 0 | 4 | 0 | 0 | 496 | 500 |
| 0.3 | 0 | 11 | 0 | 0 | 500 | 502 |
| 0.4 | 5 | 514 | 0 | 0 | 503 | 504 |
| 0.5 | 514 | 12323 | 7 | 0 | 506 | 506 |
| 0.6 | 116883 | 179971 | 28 | 0 | 508 | 510 |
| 0.7 | 13943938 | 1828291 | 514 | 0 | 511 | 514 |
| 0.8 | 1030266667 | 14284264 | 6315 | 6 | 514 | 522 |
| 0.9 | 52493333333 | 89179521 | 60123 | 163 | 518 | 534 |
| 1 | 1972016666667 | 470333333 | 441255 | 42648 | 520 | 552 |
